# Clinical Findings and Pro-Inflammatory Cytokines in Dengue Patients in Western India: A Facility-Based Study

**DOI:** 10.1371/journal.pone.0008709

**Published:** 2010-01-14

**Authors:** D. Priyadarshini, Rajesh R. Gadia, Anuradha Tripathy, K. R. Gurukumar, Asha Bhagat, Sampada Patwardhan, Nitin Mokashi, Dhananjay Vaidya, Paresh S. Shah, D. Cecilia

**Affiliations:** 1 National Institute of Virology, Pune, India; 2 Department of General Medicine, King Edward Memorial Hospital, Pune, India; 3 Department of Microbiology, Deenanath Mangeshkar Hospital, Erandwane, Pune, India; 4 Department of Microbiology, Yashwant Rao Chauhan Memorial Hospital, Pune, India; 5 John Hopkins Medical Institutions, Baltimore, Maryland, United States of America; Karolinska Institutet, Sweden

## Abstract

**Background:**

Descriptions of dengue immunopathogenesis have largely relied on data from South-east Asia and America, while India is poorly represented. This study characterizes dengue cases from Pune, Western India, with respect to clinical profile and pro-inflammatory cytokines.

**Methodology/Principal Findings:**

In 2005, 372 clinically suspected dengue cases were tested by MAC-ELISA and RT-PCR for dengue virus (DENV) aetiology. The clinical profile was recorded at the hospital. Circulating levels of IFN-γ, TNF-α, IL-6, and IL-8 were assessed by ELISA and secondary infections were defined by IgM to IgG ratio. Statistical analysis was carried out using the SPSS 11.0 version.

Of the 372 individuals, 221 were confirmed to be dengue cases. Three serotypes, DENV-1, 2 and 3 were co-circulating and one case of dual infection was identified. Of 221 cases, 159 presented with Dengue fever (DF) and 62 with Dengue hemorrhagic fever (DHF) of which six had severe DHF and one died of shock. There was a strong association of rash, abdominal pain and conjunctival congestion with DHF. Levels of IFN-γ were higher in DF whereas IL-6 and IL-8 were higher in DHF cases (p<0.05). The mean levels of the three cytokines were higher in secondary compared to primary infections. Levels of IFN-γ and IL-8 were higher in early samples collected 2–5 days after onset than late samples collected 6–15 days after onset. IFN-γ showed significant decreasing time trend (*p* = 0.005) and IL-8 levels showed increasing trend towards significance in DHF cases (interaction *p* = 0.059). There was a significant association of IL-8 levels with thrombocytopenia and both IFN-γ and IL-8 were positively associated with alanine transaminase levels.

**Conclusions/Significance:**

Rash, abdominal pain and conjunctival congestion could be prognostic symptoms for DHF. High levels of IL-6 and IL-8 were shown to associate with DHF. The time trend of IFN-γ and IL-8 levels had greater significance than absolute values in DHF pathogenesis.

## Introduction

Dengue is caused by infection with any of the four closely related serotypes of dengue virus (DENV) transmitted by *Aedes aegypti*. Two-thirds of the world's population is at risk, and about 50 million infections occur worldwide every year with a mortality that can vary from <1% to 20% depending on the quality of treatment [1]. Dengue is an emergent disease in India with 5,000–10,000 cases reported per year [2].

The clinical presentations of dengue vary from the self- resolving dengue fever (DF) to the more severe dengue hemorrhagic fever (DHF) or dengue shock syndrome (DSS). Immunity to one serotype does not provide protection against other serotypes [3]. The more severe form, DHF occurs mostly in individuals who acquire a heterotypic secondary infection [4].

The severity of dengue varies with age of infected individuals [5]–[6], the infecting DENV serotype/genotype [7]–[8], the immune status [9], and the genetic makeup of the population [10]. Humoral immunity was considered as the major contributing factor, supported by demonstration of antibody dependent enhancement (ADE) in vitro [11], and sero-epidemiological studies [12], [13], [14]. Later, cytokines and memory T cells [9] and recently T regulatory cells [15] have also been implicated in disease pathogenesis.

Several groups have reported increased levels of inflammatory cytokines, i.e. IFN-γ, TNF- α, IL-6 in DHF patients. Most of these reports are based on studies in infants/children from South-East Asia [16]–[20] and Tahiti [21]. In contrast, higher levels of IFN-γ, TNF-α and IL-6 have been reported in adult DF patients from Brazil [22] and India [23], [24]. Increased IL-8 levels have been associated with DHF and DSS in both adults and children [25], [26]. The significance of circulating levels of cytokines as inflammatory mediators in dengue patients is controversial and difficult to interpret [27]. This is probably because there is variation in the time of collection of samples, the age of the patients, the clinical presentation of the cases and the genetic population. Only few studies have correlated cytokine levels with day of illness [17], [21], [25]. The present study was carried out in Pune, Western India, endemic to dengue. We assessed the levels of inflammatory mediators- IFN-γ, TNF-α, IL-6 and IL-8 in well characterized dengue patients in context to their clinical presentation combined with laboratory findings, the day of illness and the immune status of the patient.

## Methods

### Ethics statement

This study was approved by the National Institute of Virology Human Ethics Committee on the basis of the guidelines laid down by the Indian Council of Medical Research. Informed consent was not obtained earlier as it is not required for specimens for viral diagnosis. As the samples have already been collected, there is no risk to the subjects except the privacy and confidentiality of the patients, which is being taken care of by using indirect identifiers and anonymous specimens without making any reference to identifying information of the patients. Waiver of the informed consent was granted by the committee on the basis of “Use of left-over specimens after clinical investigation” under the Indian Council of Medical Research Guidelines 2006.

### Clinical Samples

Patients with dengue-like illness presenting to any of three different hospitals located in three different areas of Pune - King Edward Memorial Hospital (KEMH), Yashwantrao Chavan Memorial Hospital (YCMH), or Deenanath Mangeshkar Hospital (DMH), between July and December 2005, were included in the study. From July to December 2005, our staff visited the three hospitals, every day to bring samples collected by the hospital to National Institute of Virology (NIV) for diagnosis. Patients with dengue like illness were included; those with parasitic and respiratory infections were excluded by the clinicians. Blood samples were transported on ice, plasma was separated and aliquoted. One aliquot was used for MAC-ELISA and RT-PCR and the leftover aliquot was frozen at −80°C.

Of the 372 suspected dengue patients, 40 attended the out patient department, and 332 were hospitalised. The individuals were tested for the presence of DENV-specific IgM and/or viral RNA and those positive by either of the two tests were considered as dengue patients. The clinical presentations of the patients were recorded by the clinicians of the respective hospitals, and abstracted by chart review. Patients with fever, headache, myalgia, retro-orbital pain, and rash were defined as DF. DHF patients were categorized by the presence of at least two of the DHF defining criteria of the World Health Organization [28]: hemorrhagic tendencies/manifestations, thrombocytopenia, and evidence of plasma leakage.

For each patient, the day of onset of fever was designated as the first day of illness. Plasma samples collected on 2–5 days post illness was designated as ‘early’ and 6–15 days post illness as ‘late’. Samples from 45 age-sex matched healthy blood donors were included as negative controls.

Haemoglobin level, total leukocyte count (TLC), packed cell volume (PCV), platelet count, estimation of levels of aspartate aminotransferase (AST), alanine transferase (ALT), alkaline phosphatase (ALP) and bilirubin were carried out at KEMH and DMH for 95 patients. Data were obtained from the patients' clinical charts.

### Laboratory diagnosis

Blood samples from all 372 cases were tested in the laboratory for dengue aetiology. The in-house National Institute of Virology (NIV) IgM capture ELISA kit was used to detect DENV-specific IgM [29]. A known positive (P) and a known negative (N) serum control were used in every test. A sample showing an OD value >2.1 times the P/N ratio was considered positive.

Samples were tested for presence of viral RNA and serotyped by the multiplex nested RT-PCR. RNA was extracted from 140-µl serum/plasma using a viral RNA extraction kit (Qiagen Sciences, Valencia, CA, USA). Group specific primers were used in the first cycle of the RT-PCR and serotype-specific primers were used in the second cycle as described previously [30]. The amplicons were sequenced for confirming the serotype detected using Big Dye terminator kit (Applied Biosystems, Foster city, CA, USA).

Classification of primary and secondary DENV infection was done using IgM and IgG capture ELISA kits (Panbio, Windsor, Australia) for a subset of 123 samples depending on availability. IgG levels of >22 units (defined by the manufacturers) or ratio of IgM to IgG of <1.78 indicated secondary infection [31].

### Cytokine estimation

Estimation of four cytokines, Interferon-γ (IFN-γ), Tumor necrosis factor-α (TNF-α), Interleukin-6 (IL-6) and Interleukin-8 (IL-8) in the plasma samples was done using the human cytokine ELISA kits (Opt EIA Sets, BD Biosciences, USA) for the 221 confirmed dengue cases and 45 apparently healthy controls. Standards were included in each assay and the standard curve was used for estimation of cytokine concentration (in pg/ml) by regression analysis. The detection limits of the kit for IFN- γ, TNF-α, IL-6, and IL-8 were 4.6, 7.8, 4.6, and 3.1 pg/ml respectively. The mean level of the cytokines in plasma samples of the healthy individuals plus 2 S.D. was used as the cut-off.

### Data and Statistical analysis

Descriptive data were expressed as mean with SEM levels or number (percent) of cases. The chi-square test (with Yates correction, wherever required) was used to examine differences in demographic and clinical characteristics of the study group. The values of cytokine levels were corrected by adding 1 in order to obtain log values for statistical analysis. Group comparison data for cytokines is presented as mean with SEM levels. Univariate ANOVA was carried out and if significance found, post-hoc (Tukey) test was used for defining differences within groups. To detect the significant differences between patient groups with respect to post onset days, general linear model (GLM) was used, including the interaction term. Pearson correlation was used to study the correlation between the cytokine levels and clinical findings. A *p*≤0.05 was considered significant. All the statistical analyses of the data were done using the SPSS 11.0 program and graphs plotted using GraphPad Prism 5.1 (San Diego California, USA).

## Results

### Patients' characteristics

Of the 372 dengue suspected cases, 221 (59%) were confirmed to be dengue by laboratory tests, 98% of who were hospitalized (n = 217). A total of 195 patients were positive for dengue-specific IgM and 32 tested positive for DENV-RNA. Six samples were positive for both viral RNA and IgM. Table 1 presents the demographic characteristics, serotypes detected and disease categories for the 221 cases.

**Table 1:** Demographics, Serotypes detected and disease categories.

Category	Subcategory	Number of cases (%)
Total number of dengue cases		221
Median Age (years)		24
**Sex**	Males	180 (81)
	Females	41 (19)
RT PCR positive cases		32 (14)
**Serotypes detected**		
	DENV-1	16 (50)
	DENV-2	10 (31)
	DENV-3	5 (16)
	DENV-2/3	1 (3)
Dengue fever		159 (71.9)
	With thrombocytopenia	81 (51)
Dengue hemorrhagic fever		62 (28)
	Severe cases	7 (11)

**Figure 1:**
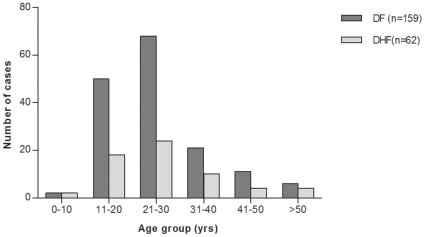
Age-wise distribution of DF and DHF cases. Number of dengue cases plotted according to age – groups.

The age distribution of the dengue patients is shown in Figure 1, with the median age being 24 years (range 1–64 years). There was a larger representation of males as indicated by the male: female ratio of 4.4∶1. The higher representation of males was reflected in the fever cases attending the hospitals (data not shown). Of the three serotypes (DENV-1, 2 and 3), DENV-1 was predominant (50%).

Based on clinical features, 71.9% (n = 159) of the patients were classified as DF, 50% of them had thrombocytopenia without any bleeding. Presence of any two of the DHF defining criteria by WHO, categorized 62 patients as DHF cases. Seven patients were defined as severe DHF (grades III/IV) with signs of circulatory failure. One fatal patient had severe thrombocytopenia, plasma leakage and circulatory failure (Grade IV). The distribution of DF and DHF cases did not differ by age (Fig. 1). An 11-year-old boy, who presented with DF was found to have a dual infection with DENV-2 and DENV-3, (Table 1).

To identify possible predictors for DHF, the clinical symptoms presented by the 221 confirmed cases common to DF and DHF were analyzed in context to disease category (Table 2). Fever with chills, headache, myalgia and nausea/vomiting were reported equally by DF and DHF patients. Joint pain, retro-orbital pain and itching were observed in a significantly larger number of DF cases (*p*<0.05, χ^2^ test). Abdominal pain, maculopapular rash/petechiae and conjunctival congestion were found to be more prevalent in DHF cases (*p*<0.05, χ^2^ test). The percentage of DHF patients with thrombocytopenia (92%) was significantly higher than the percentage of DF patients (51%) (*p*<0.01, χ^2^ test).

**Table 2:** Clinical signs/symptoms in DF and DHF patients.

Signs/Symptoms	DF cases (n = 159), n(%)	DHF cases (n = 62), n (%)	Comparison between DHF and DF	*p*- value
Myalgia	101 (63.5)	33(53.2)	-	0.20
Headache	101 (63.5)	33(53.2)	-	0.20
Nausea/Vomiting	90 (56.6)	44 (70.9)	-	0.07
Cough	15 (10.8)	4 (4.9)	-	0.20
Sore throat	8(5)	3(4.8)	-	0.95
Itching	29 (18.2),	3 (4.8)	**DF>DHF**	**0.01**
Joint pain	42 (19.5)	3 (4.8)	**DF>DHF**	**<0.001**
Retro-orbital pain	34 (21.3)	2(3.23)	**DF>DHF**	**<0.001**
Abdominal pain	29(18.2)	31 (50)	**DHF>DF**	**<0.001**
Petechiae/Rash	31 (19.4)	26 (29.6)	**DHF>DF**	**0.001**
Conjunctival congestion	7 (4.4)	14 (22.6)	**DHF>DF**	**<0.001**
Thrombocytopenia	81 (50.9)	87 (91.9)	**DHF>DF**	**<0.001**

Comparison between DF and DHF patients done by chi-square test, *p*-value <0.05 considered significant.

The metabolic parameters and hematological profiles available for 95 samples (42 DF cases, 53 DHF cases) were also analyzed in the context of DF/DHF (Table 3). An increase in the hematocrit (Hct) value, ≥20% above the area specific cut-off levels, which is the defining criteria for DHF by WHO, was observed in 17 DHF patients (35.4%) and an increase of ≥10–19% was observed in 11 DHF patients (21%), which has also been considered as abnormal by Nyugen *et al.*, [32]. Both hemoconcentration and leucopenia were observed in a larger number of DHF cases compared to DF, but the difference was not statistically significant. Elevated levels of AST and ALT were seen in both DHF and DF patients.

**Table 3:** Laboratory findings in DF and DHF.

Variable	Normal range	DF	DHF	*p* value
Peak hematocrit, mean %±SD	36%	39.9±4.3	39.9±5.5	
Increase in hematocrit, no of cases, (%)		8/38 (21)	17/48 (35.4)	0.22
Leucocyte count, mean cells×10^3^±SD	4000–11000/cu mm	4.1±2.3	4.8±2.7	
No. of cases with leucopenia (%)		21/39 (53.8)	25/53 (47.1)	0.67
AST levels, mean±SD	Upto 30U/l	133±107.1	178.3±190.4	
No. of cases with Increased levels of AST		29/30 (96.6)	45/46 (97.8)	0.75
ALT levels, mean±SD	Upto 40U/l	105±87.1	168.1±243.2	
No. of cases with increased levels of ALT (%)		25/32 (78.1)	39/49 (79.5)	0.90
Alkaline phosphatase levels, mean±SD	Upto 275 U/l	239.4±231	259.7±223.7	
No.of cases with increased levels of ALP (%)		9/28(32.1)	10/41 (24.3)	0.66
Bilirubin levels	<1mg%	0.81±0.68	11.8±73.7	
No.of cases with>1 mg%		4/30(13.3)	9/46 (19.5)	0.69

Cases compared using chi-square test, *p*- value <0.05 considered significant.

In the total 62 DHF cases, gastrointestinal bleeding, manifested by melena or hematemesis was reported in 32 (51.6%) cases. Hematuria observed in six (9.7%), gum bleeding in six (9.7%), conjunctival hemorrhage in four (6.4%) and epistaxis in two (3.2%) patients were less common. Splenomegaly and hepatomegaly was evidenced in 11 (17.7%) and in 7 (11.3%) cases respectively. Plasma leakage was observed in 23 (37%) patients –either as ascites (n = 22) and/or as pleural effusion (n = 16).

### Levels of cytokines in dengue patients

**Figure 2:**
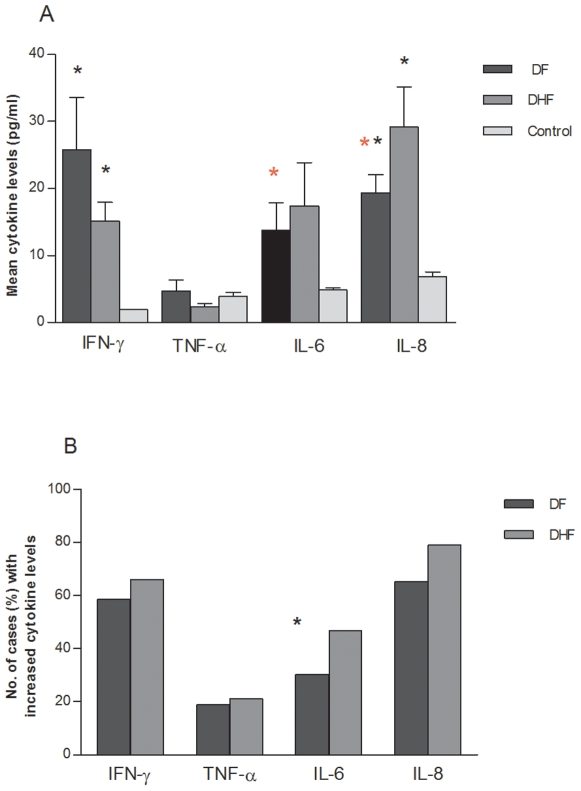
Levels of IFN-γ, TNF-α, IL-6 and IL-8 in DF and DHF patients. A. Mean with SEM levels (pg/ml) of cytokines. B. Number (%) of cases with increased (above cut-off) values of cytokines in DF/DHF. Analysis of variance was carried out for log transformed values of each cytokine and differences in groups analysed by using Tukey test.*p-value <0.05 when compared with controls. *p-value <0.05 when compared between DF and DHF. Comparison between number of cases for DF and DHF done using chi-square test. *p*-value <0.05 is shown.

The circulating levels of four cytokines, IFN-γ, TNF-α, IL-6 and IL-8 were assessed in the 221 confirmed dengue cases (Fig. 2).

### IFN-γ levels

A mean level of 1.3 pg/ml of IFN-γ was detected in healthy controls. Using 3.8 pg/ml as cut off, IFN-γ was found to be significantly higher (*p*<0.001) in dengue patients (both DF and DHF), with a range of 0.9 to 859.7 pg/ml. The mean level of IFN-γ in DF cases was higher compared to the mean level in DHF patients (Fig. 2A). The number of cases showing increased IFN-γ levels was larger in the DHF category (66.1%, n = 41) as compared to DF cases (58.4%, n = 93) (Fig. 2B).

### TNF-α levels

The mean level of TNF-α in healthy controls was 3 pg/ml and the cut off was 5.5 pg/ml. The concentration of TNF-α ranged from 0 to 184 pg/ml in dengue patients (Fig. 2A,B). The mean values of DF and DHF were lower than that of healthy controls (*p*>0.05).

### IL-6 levels

The mean level of IL-6 in healthy controls was 4.8 pg/ml and 8.9 pg/ml was the cut off value. The values in dengue cases ranged from 0.13 to 417 pg/ml. The levels of IL-6 in all dengue cases were higher than healthy controls, but not statistically significant (p>0.05). However, with disease categorization, the levels of IL-6 in DHF were higher than in DF patients (p = 0.02, Tukey test). DHF cases also showed a higher mean level (Fig. 2A) and a larger number of cases with high levels (47%) (Fig. 2B).

### IL-8 levels

The level of IL-8 in healthy controls was variable ranging from 1 to 25 pg/ml, resulting in a high cut off value of 16.26 pg/ml. The range of values observed in dengue patients was 1 to 355.3 pg/ml and on comparison with controls significantly higher (*p*<0.001). The levels in DHF were significantly higher than in DF cases (*p*<0.001, Tukey test). The mean value of IL-8 (Fig. 2A) and the number of cases with increased levels (79%) were also higher in DHF group (Fig. 2B).

In summary, higher levels of IFN-γ, IL-6 and IL-8 were observed in dengue cases compared to healthy controls. Furthermore, the levels of IL-6 and IL-8 were significantly higher in DHF cases as compared to DF cases.

### Cytokine levels on different post onset days

We further investigated whether it was the early (2–5 days of illness) or late (6–15 days of illness) cytokine response, which contributed to differences observed between DF and DHF cases using the interaction term in the general linear model of analysis. The IFN-γ levels showed a time trend (*p*<0.001), which did not differ by group. The levels of IL-6 did not show a time trend. Levels of IL-8 on the other hand showed a time trend that differed for DF and DHF (*p* = 0.016).

When the cytokine levels were analysed for time trend for individual DF or DHF groups we observed that the levels of IFN-γ showed significant decreasing trend (*p* = 0.005) and IL-8 levels showed increasing trend towards significance in DHF cases (interaction *p* = 0.059).

**Figure 3:**
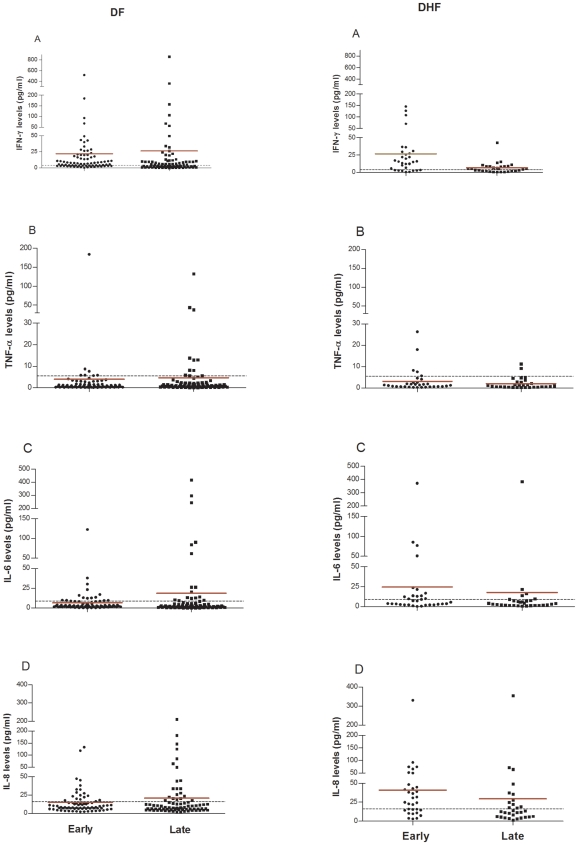
Cytokine levels (pg/ml) in early and late days of illness in DF and DHF patients. Scatter plot of levels of cytokine (pg/ml) in early (2–5) and late (6–15) days of illness. A) IFN-γ, B) TNF-α, C) IL-6 and D) IL-8. The two panels show DF and DHF cases. The mean levels are indicated with the red line. The cut-off (mean levels in healthy controls+2SD) for each cytokine is shown with a dashed line.

This prompted us to compare the levels of cytokines in DF and DHF cases during the early and late period of infection (Fig. 3). The levels of IL-6 (*p* = 0.005) and IL-8 (*p*<0.001) were found to be significantly higher in DHF cases as compared to DF in the early phase while the difference was not significant in late phase.

### Effect of immune status of the host

Based on the criteria of 22 IgG units qualifying a secondary infection, 62% of DF cases and 38% of DHF cases were classified as secondary, which is contrary to the earlier findings of larger number of secondary cases in DHF [9]. Therefore the criteria of IgM/IgG ratio of <1.78 to be indicative of secondary infection [31] was used. The number of secondary cases increased in both categories, 74% in DF cases and 85.7% in DHF cases.

**Figure 4:**
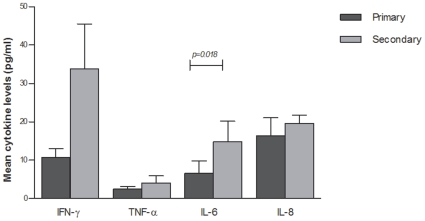
Cytokine levels in primary and secondary DENV infections. Mean with SEM levels of four cytokines (pg/ml) in primary and secondary infections. Patients with IgM/IgG ≥1.78 had primary infections; IgM/IgG<1.78 had secondary infections. p-value calculated by analysis of variance of log transformed levels of cytokines. p<0.05 considered significant.

The levels of the four cytokines were therefore, analysed in context to primary versus secondary infections defined by IgM/IgG ratio (Fig. 4). The mean levels of all four cytokines were higher in secondary cases compared to primary cases. Higher levels of IL-6 (*p* = 0.018) and IL-8 (*p* = 0.06) were observed in secondary cases compared to primary dengue cases.

### Relationship between cytokines and clinical parameters

**Figure 5:**
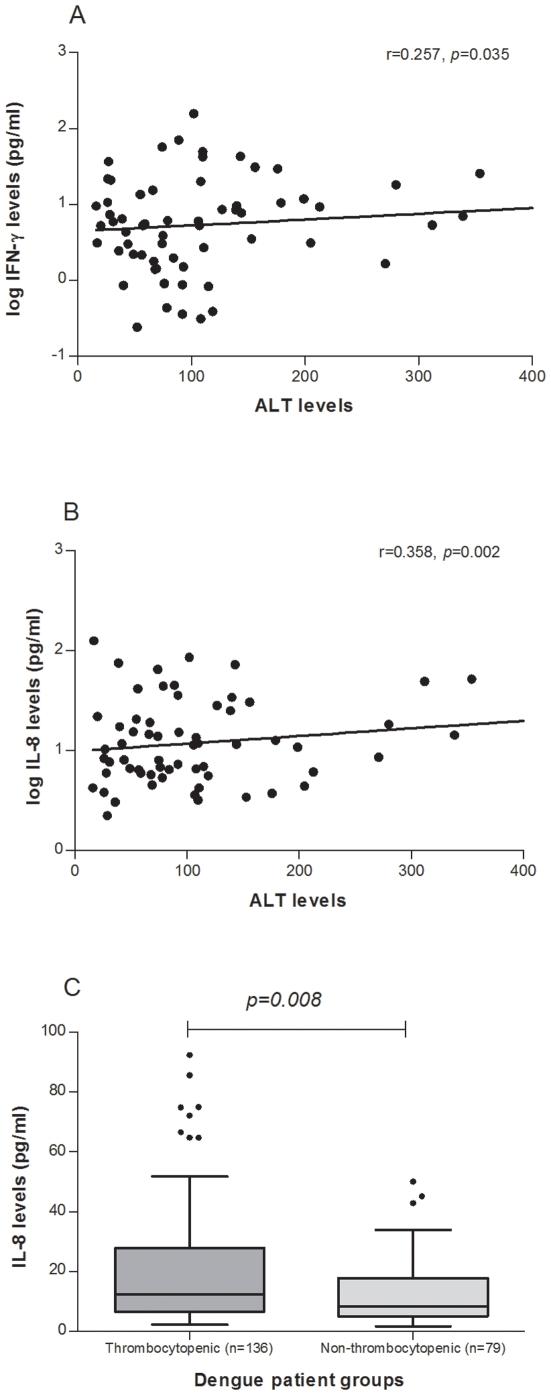
Association of cytokine levels with clinical parameters of dengue patients. A. Association of levels of IFN-γ with ALT levels. B. Association of levels of IL-8 with ALT levels. C. Tukey box-whisker plot with median, range with the upper and lower quartiles and outliers of IL-8 levels in thrombocytopenic (platelet count <100,000) and non- thrombocytopenic (platelet count >100,000) dengue patients. A & B] Pearson correlation used for analysis, *p*<0.05 considered significant. C] *p*-value calculated by ANOVA, *p*<0.05 considered significant. Outliers showed as dots and those above the axis limit not shown in graph but included in analysis.

We then analysed the association between increased levels of IFN-γ, IL-6 and IL-8 with parameters indicative of cell damage, thrombocytopenia, a correlate of platelet destruction, increased hematocrit values, pleural effusion/ascites, indicative of endothelial cell dysfunction and raised AST/ALT, suggestive of liver damage. Increased levels of IFN-γ were observed in a larger number of cases with thrombocytopenia (58.2%), increased hematocrit (64%) and pleural effusion/ascites (50%) but the difference was not significant when compared to levels in patients without the manifestations. However, the association of IFN-γ levels with the ALT levels was significant (r = 0.257, *p* = 0.035) (Fig. 5A). Increased levels of IL-6 were observed in 65% of the patients who showed pleural effusion/ascites. IL-8 levels showed weak association with ALT levels (r = 0.358, *p* = 0.002), (Fig. 5B). The levels of IL-8 in dengue patients with thrombocytopenia were significantly higher than in the patients that showed normal platelet count (*p* = 0.008, Tukey test), (Fig. 5C).

## Discussion

In the present study, we have assessed the clinical and cytokine profile of dengue cases in Pune in 2005. The study area is located in Maharashtra, Western India, known to be endemic to dengue with 600–800 cases occurring annually [2]. The reports so far from India have mostly described DHF outbreaks in Delhi [33], [34], [35]. In Pune, the representation of adults (72%) was higher and the representation of severe cases was very low. Only 27% of the cases could be classified as DHF, lower than that reported in Delhi. There were multiple serotypes co-circulating, DENV-1 being the predominant serotype followed by DENV-2 and DENV-3. Co-circulation of multiple serotypes is now being commonly reported [36]–[37] and might be responsible for the increase in occurrence of DHF. One individual who evidenced dual infection with DENV-2 and 3 had mild disease; similar to a report from Brazil [38] suggesting that simultaneous infection with two serotypes does not exacerbate the disease.

Of the symptoms common to dengue infected patients, joint pain, retro-orbital pain, and itching were seen in a significantly higher proportion of DF cases. On the other hand rash, abdominal pain and conjunctival congestion showed a positive association with DHF and could be used as prognostic symptoms for DHF. Blanching rash and abdominal pain are already considered as strong associates of DHF [28]. Gastrointestinal (GI) bleeding manifested by melena and hemetemesis was the most common hemorrhagic manifestation in DHF, similar to the observations in adult dengue cases from Thailand [5] and Srilanka [6]. Liver involvement, which has been well documented in DHF [39], was evidenced in 85% of the DHF patients tested.

Strict application of WHO criteria (which was formulated based on DHF in children in South-East Asia) was found to miss many cases of DHF [40] as shown by studies on adult population in Nicaragua [41] and in Thailand [42]. The hospitals participating in this study reported a difficulty in interpreting the tourniquet test, which was therefore not included as distinguishing criteria for DHF. Earlier reports from India found <40% DHF cases positive for the tourniquet test [43], [44]. Thus, presence of any two of the DHF classifying criteria by WHO - hemorrhage, hemoconcentration, plasma leakage and thrombocytopenia or circulatory collapse were used to classify DHF. Thrombocytopenia was also detected in 48% of DF cases. We observed a fairly large representation of secondary cases in both DF (75%) and DHF (86%) categories. It is possible that with multiple circulating strains in an endemic area, many individuals could acquire two infections in a lifetime without ever experiencing severe dengue infection. At the same time, one can also question whether the non-inclusion of the tourniquet test resulted in inclusion of DHF I cases into the DF category or is it that DENV still maintains its moderate virulence in India in an endemic situation.

Assessment of cytokines and their correlation with disease revealed that three cytokines, IFN-γ, IL-6, and IL-8 were significantly elevated in dengue cases as compared to healthy controls. The levels of the four cytokines were analysed in context of the clinical presentation, the time of sampling, and the immune status of the DF and DHF cases to understand their relevance to disease pathogenesis.

The levels of IFN-γ were higher in DF cases compared to DHF, as reported earlier [22]–[24] but a higher proportion of DHF patients had elevated IFN-γ levels, similar to findings of Kurane *et al.*, [16]. Analysing the data in context of day of illness, a time trend existed for IFN-γ and in DHF cases the trend from high in the early phase to low levels in the late phase was significant. The early IFN-γ response is believed to be important in DHF, peak levels being found to precede the onset of plasma leakage [17]. In our study, increased levels of IFN-γ were found in larger number of DHF cases with plasma leakage and showed weak association with raised ALT levels, suggesting a potential role for IFN-γ in dengue pathogenesis.

In contrast to other studies in which elevated levels of TNF-α were reported in patients with DHF and DSS [17], [45], [46], the levels of TNF-α observed in our study were very low. This was probably because the sampling was not early enough and the severity of disease was milder with very few cases of DHF III/IV. In addition, it is possible that genetic polymorphism in the TNF-α gene may be a contributing factor [47], [48].

Comparison of the levels of IL-6 in DF versus DHF cases revealed that the DHF cases had significantly higher values. IL-6 was also associated with presence of pleural effusion/ascites in DHF, consistent with an earlier report [19] but not with thrombocytopenia or increased ALT.

The level of IL-8 seemed more relevant to DHF pathogenesis, not only was it significantly higher in DHF compared to DF but also correlated with thrombocytopenia and raised ALT. Recent study has shown up-regulation of the genes expressing pro-inflammatory cytokines including IL-6 and IL-8 in HepG2 cells *in vitro.* [49]. Increased levels of IL-8 have been associated with plasma leakage [18], [26], [50], [51].

Unique to our study, a time trend for IL-8 levels was found in early to late post-onset day of illness in DHF cases. In addition, together with IL-6, the early levels of IL-8 were higher in DHF as compared to DF cases.

Higher levels of all four cytokines were observed in secondary infections as compared to primary infections. This supports the relevance of enhanced cytokine secretion in the T-cell mediated immunopathology of secondary DENV infection [9].

### Conclusion

Our study has provided the clinical picture of dengue cases in Pune, along with the cytokine responses. DHF is seen in 27% of the cases with low severity despite the circulation of multiple serotypes. It suggests that abdominal pain, rash and conjunctival congestion could be considered as warning symptoms for development of DHF and confirms the hepatic involvement in adult DHF cases. Higher levels of IL-6 and IL-8 early in course of infection may be prognostic markers for progression to DHF and seem to play a role in the disease pathogenesis.
